# Retraction: Increasing interpersonal trust through divergent thinking

**DOI:** 10.3389/fpsyg.2024.1384407

**Published:** 2024-02-15

**Authors:** Frontiers Editorial Office

The journal retracts the 06 June 2014 article cited above.

This retraction follows the recommendations of an investigation by Leiden University's Committee on Scientific Integrity which found that the paper contained data manipulation that invalidates the study's findings. The retraction has been approved by the Chief Executive Editor of Frontiers. Authors S. van de Groep, E. W. de Kwaadsteniet and R. Sellaro agree to this retraction. Authors L. S. Colzato and B. Hommel do not agree to this retraction.

